# Association of COVID-19 Risk Misperceptions With Household Isolation in the United States: Survey Study

**DOI:** 10.2196/30164

**Published:** 2021-08-30

**Authors:** Joseph A Ladapo, Jonathan T Rothwell, Christina M Ramirez

**Affiliations:** 1 Division of General Internal Medicine and Health Services Research David Geffen School of Medicine University of California, Los Angeles Los Angeles, CA United States; 2 Gallup Washington, DC United States; 3 Department of Biostatistics Fielding School of Public Health University of California, Los Angeles Los Angeles, CA United States

**Keywords:** COVID-19, pandemic, mental health, public health, isolation, loneliness, guideline, risk, perception, United States, health risk, well-being

## Abstract

**Background:**

Adverse mental and emotional health outcomes are increasingly recognized as a public health challenge associated with the COVID-19 pandemic.

**Objective:**

The goal of this study was to examine the association of COVID-19 risk misperceptions with self-reported household isolation, a potential risk factor for social isolation and loneliness.

**Methods:**

We analyzed data from the Franklin Templeton-Gallup Economics of Recovery Study (July to December 2020) of 24,649 US adults. We also analyzed data from the Gallup Panel (March 2020 to February 2021), which included 123,516 observations about loneliness. The primary outcome was self-reported household isolation, which we defined as a respondent having no contact or very little contact with people outside their household, analogous to quarantining.

**Results:**

From July to December 2020, 53% to 57% of respondents reported living in household isolation. Most participants reported beliefs about COVID-19 health risks that were inaccurate, and overestimation of health risk was most common. For example, while deaths in persons younger than 55 years old accounted for 7% of total US deaths, respondents estimated that this population represented 43% of deaths. Overestimating COVID-19 health risks was associated with increased self-reported household isolation, with percentage differences ranging from 5.6 to 11.8 (*P*<.001 at each time point). Characteristics associated with self-reported household isolation from the July and August 2020 surveys and persisting in the December 2020 survey included younger age (18 to 39 years), having a serious medical condition, having a household member with a serious medical condition, and identifying as a Democrat. In the Gallup Panel, self-reported household isolation was associated with a higher prevalence of loneliness.

**Conclusions:**

Pandemic-related harms to emotional and mental well-being may be attenuated by reducing risk overestimation and household isolation preferences that exceed public health guidelines.

## Introduction

Adverse mental and emotional health outcomes are increasingly recognized as a public health challenge associated with the COVID-19 pandemic. As early as March 2020, a national survey reported that 36% of US adults felt the pandemic would have a serious impact on their mental health [1]. In April 2020, another survey found that 14% of US adults reported serious psychological distress, compared to 4% during a similar time period in 2018 [2]. Rates of loneliness have also been high, with 36% of US adults—including 61% of adults aged 18 to 25 years—reporting significant loneliness in an October 2020 survey [3]. More recently, a March 2021 survey found that 48% of adults reported higher levels of stress in their lives compared to before the pandemic, and 61% reporting undesired weight changes [4].

These health sequalae of the COVID-19 pandemic are multifactorial, and social isolation is likely an important contributor [5,6]. Because of physical distancing mandates, quarantines, and fear of illness, a substantial proportion of Americans have limited their physical contact with others outside of their household. This trend has likely contributed to social isolation and loneliness. Household isolation is analogous to quarantining, and research has shown that quarantining is a risk factor for a variety of adverse mental and emotional health outcomes, including increased stress, anxiety, depression, fear, and detachment from other people [5,7].

The US Centers for Disease Control and Prevention (CDC) recently recommended that researchers examine drivers of adverse mental health during the COVID-19 pandemic [8]. One driver that has received little attention is the role that COVID-19 risk misperceptions may play in the behavioral decision to limit physical contact with others. While COVID-19 risk perceptions have been associated with protective health behaviors [9], they may lead to suboptimal behavioral choices, if individuals substantially overestimate or underestimate risk [10,11]. Overestimation, in particular, is of concern in the context of mental and emotional health and well-being because it tends to amplify social isolation and reduce contact with others. Using survey data from the Franklin Templeton-Gallup Economics of Recovery Study, we assessed the association of COVID-19 risk misperceptions with self-reported household isolation. Our findings are relevant to policy measures to reduce COVID-19–related social isolation and may inform the management of future epidemics and pandemics.

## Methods

### Data

We used data from the Franklin Templeton-Gallup Economics of Recovery Study, a self-administered web survey from an opt-in sample provided by Dynata of 24,649 US adults, aged 18 years and older, of whom 10,419 participated during more than one survey time point. The survey was conducted during the following time points: July 2 to 14, August 3 to 11, September 4 to 13, October 1 to 9, November 2 to 6, and December 1 to 7, 2020. Gallup weighted the obtained sample to correct for nonresponse and construct a nationally representative population. Nonresponse adjustments were made by adjusting the sample to match the national demographics of gender, age, race and ethnicity, region, educational level, marital status, and employment status. Demographic weighting targets were based on the Census Bureau’s 2018 data release of the American Community Survey and the Current Population Survey (February 2020).

We also supplemented this survey with data from the Gallup Panel, a research panel that is representative of the US adult population and includes approximately 100,000 members. Gallup fielded the COVID-19 tracking survey on March 13, 2020, and collected approximately 1000 responses daily until April 26, 2020, when the sample declined to approximately 500 responses daily. The Gallup Panel’s COVID-19 Tracking Survey includes information about self-reported household isolation and loneliness, with 123,516 observations from March 24, 2020, to February 21, 2021. This study was exempt from institutional review board review according to policies of the UCLA (University of California, Los Angeles) Office of the Human Research Protection Program.

### Primary Measures

We assessed self-reported household isolation by asking participants about the degree of in-person contact outside their household that they had over the past 24 hours. We considered a participant to be isolated if they reported being *completely isolated* (no contact) or *mostly isolated* (very little contact) from people outside their household. We assessed loneliness in the Gallup Panel survey by asking participants, “Did you experience the following feelings during a lot of the day yesterday?” Loneliness and other emotional experiences were included as response options. The specific questions and respondent options are provided in Multimedia Appendix 1.

### Perceptions of COVID-19 Health Risks

We evaluated perceptions of COVID-19 health risks using multiple questions. In July and August, respondents were asked about the percentage of all US COVID-19 deaths that fell into the following age strata: age 24 years and below, age 25 to 34 years, age 35 to 44 years, age 45 to 54 years, age 55 to 64 years, and age 65 years and older. We assessed misperceptions using the reported proportion of deaths attributable to persons under the age of 55 years because most deaths from COVID-19 have occurred in persons older than 55 years. Perceptions about age-related COVID-19 health risks were assessed in September and October 2020 with an analogous question about the age distribution of COVID-19 hospitalizations.

In the November 2020 survey, respondents were asked what percentage of patients hospitalized with COVID-19 died. In the December 2020 survey, respondents were asked what percentage of patients infected with COVID-19 required hospitalization.

### Estimation of Actual COVID-19 Health Risks

CDC data were used to estimate the proportion of deaths from COVID-19 by age. Data from the COVID-19–Associated Hospitalization Surveillance Network (COVID-NET) was used to estimate hospitalizations by age [12]. Because COVID-NET data on hospitalizations were reported using different age strata than those provided to survey respondents, we adjusted these data using simple proportional methods. Specifically, we multiplied hospitalizations reported in COVID-NET by the proportion of years in a corresponding age stratum in order to recategorize hospitalizations into different age strata. The likelihood of hospitalization after infection was estimated to be approximately 5% as reported by Reese and colleagues from the CDC estimate that there were 52.9 million infections from February to September 2020 and 2.4 million hospitalizations, implying a hospitalization rate of 4.5% [13]. Their method accounted for underreporting. We estimated the likelihood of death among patients hospitalized for COVID-19 to be 12% based on an analysis of 38,517 hospitalized patients from January 1, 2020, to June 30, 2020 [14].

### Other Measures

In each wave, survey respondents were asked whether they or a household member had a comorbidity that increased the risk of severe COVID-19 illness. Respondents also reported sociodemographic characteristics, household income, and preferences for political parties. Per capita deaths from COVID-19 in each US county from March 1 until December 1 were assessed using CDC data [15].

### Analyses of Survey Data

Descriptive analyses of respondents’ characteristics were performed using data from July, August, and December 2020. The July and August surveys were combined for analyses because questions about risk perception were identical between those two time points. The September and October surveys were similarly combined. We used multivariable logistic regression analyses to examine the relationship between misperceptions about COVID-19 health risks and social isolation. Results from these models were presented as predictive margins, in which the regression models were used to estimate the marginal effect of risk overestimation, expressed as a proportion, while holding the distribution of all other covariates constant [16]. The adjusted association of respondent characteristics with social isolation was also presented using data from July and August as well as December in order to examine how behavioral patterns may have shifted over the course of the pandemic. In a secondary analysis, we used Gallup Panel data to assess the relationship between self-reported household isolation and loneliness.

Perceptions about risk were characterized as being overestimates, underestimates, or accurate estimates. To provide respondents with a reasonable degree of latitude and to account for any uncertainty in our reference estimates, we considered responses that were within 5 percentage points above or below the correct estimate as being accurate (eg, a response of 15% for estimated hospital mortality would be considered accurate because it fell within 5 percentage points of the actual rate of 12%) [14]. A range of 10 percentage points above or below was used for respondents’ estimates of the proportion of hospitalizations occurring in persons younger than 55 years old, due to the larger proportion.

We performed mean imputation from the overall sample for education (missing <1%), income (missing <1%), and whether the respondent or their family had a serious medical condition (missing <1%). We used this method instead of a more robust multiple imputation model because of the low rate of missingness. We did not report percentages as n/N values because all reported percentages were estimated using analytic weights. All analyses were performed using Stata 14 (StataCorp LP) and incorporated analytic weights to account for the effects of nonresponse.

### Data Availability

The data used in this study are available from the corresponding authors upon request and with the permission of Franklin Templeton and Gallup.

## Results

### Overview

We present descriptive characteristics of the respondents in Table 1. The mean age of the respondents was 47 years (SD 18), and 52.3% were female. The largest proportion of respondents had a household income that ranged from US $48,000 to US $89,999. Half of the respondents reported that they or a household member had a serious medical condition that increased their risk of serious illness from COVID-19.

**Table 1:** Characteristics of the study sample by time period.

Characteristics			July to August 2020: participants (N=15,014), n (%)^a^		September to October 2020: participants (N=10,019), n (%)^a^		November 2020: participants (N=5026), n (%)^a^		December 2020: participants (N=5009), n (%)^a^
**Age group (years)**									
	18-39	5333 (37.5)		3665 (37.7)		1966 (38.7)		1749 (38.3)	
	40-64	6347 (41.3)		4140 (41.2)		1978 (40.4)		2148 (40.6)	
	≥65	3334 (21.2)		2214 (21.1)		1082 (20.9)		1112 (21.1)	
**Female**									
	Yes	8032 (52.3)		5430 (52.2)		2711 (51.9)		2733 (51.9)	
	No	6982 (47.8)		4589 (48.0)		2315 (48.2)		2276 (48.3)	
**Race or ethnic group**									
	White	9512 (63.4)		6465 (63.7)		3206 (63.7)		3228 (63.6)	
	Black	1909 (12.4)		1289 (12.3)		694 (12.3)		680 (12.4)	
	Hispanic	2491 (16.4)		1503 (16.0)		788 (16.0)		720 (15.8)	
	Other or unknown	1102 (7.8)		762 (8.1)		338 (8.1)		381 (8.2)	
**Educational level**									
	8th grade or some high school	328 (4.0)		274 (4.0)		165 (4.6)		106 (3.4)	
	High school graduate	2889 (36.7)		2268 (37.0)		1155 (36.5)		1173 (38.1)	
	Some college or college graduate	11,797 (59.5)		7477 (59.1)		3706 (59.0)		3730 (58.6)	
**Serious medical condition**									
	No	7708 (51.8)		5099 (51.0)		2427 (49.1)		2414 (48.2)	
	Yes, in respondent	3843 (26.1)		2465 (25.1)		1307 (26.4)		1211 (24.8)	
	Yes, in household member	2324 (15.3)		1652 (16.6)		844 (16.6)		890 (17.9)	
	Yes, in respondent and household member	1139 (7.1)		803 (7.5)		448 (8.3)		494 (9.4)	
**Political preference**									
	Democrat	5483 (36.1)		3763 (37.3)		1940 (37.9)		1990 (38.4)	
	Republican	4539 (31.4)		3185 (32.1)		1686 (34.2)		1545 (31.2)	
	Independent	4155 (27.1)		2594 (25.8)		1190 (23.6)		1232 (24.9)	
	Other or unknown party	837 (6.1)		477 (4.9)		210 (4.5)		242 (5.7)	
**Household income (US $)**									
	<24,000	2466 (19.1)		1922 (20.7)		1009 (20.6)		931 (20.4)	
	24,000-47,999	2825 (22.2)		2146 (24.4)		1078 (25.3)		1152 (26.1)	
	48,000-89,999	4519 (30.3)		2990 (29.5)		1456 (29.3)		1546 (30.4)	
	≥90,000	5204 (28.4)		2961 (25.4)		1483 (24.8)		1380 (23.1)	
**Married**									
	Yes	8018 (48.4)		5124 (48.0)		2652 (47.8)		2567 (47.6)	
	No	6996 (51.9)		4895 (52.2)		2374 (52.3)		2442 (52.5)	
**Live in rural area**									
	Yes	1262 (9.4)		982 (10.3)		527 (11.3)		474 (10.2)	
	No	13,752 (90.6)		9037 (89.8)		4499 (88.8)		4535 (89.9)	

^a^Percentages are based on analytic weights.

### Misperceptions About COVID-19 Health Risks

**Figure 1:**
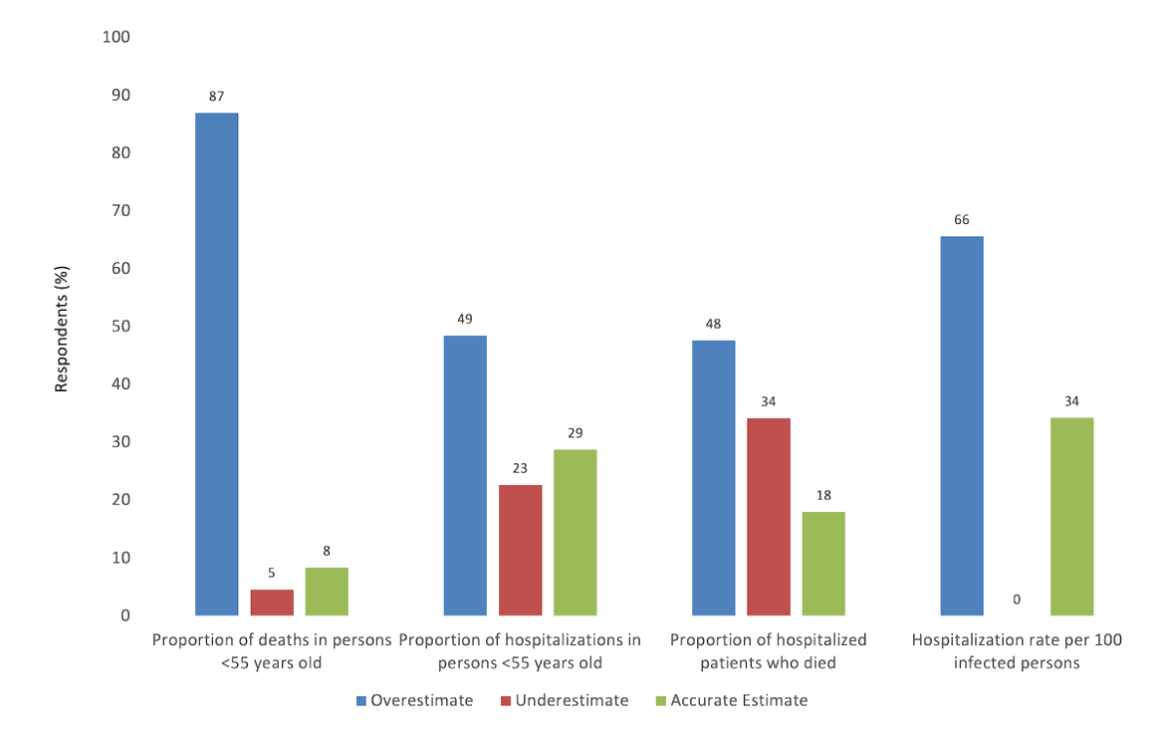
Comparison of respondents' perceived risk versus actual risk associated with COVID-19 illness.

Most participants held beliefs about COVID-19 health risks that were inaccurate (Figure 1). Overestimation of health risk, rather than underestimation, was the most common type of inaccuracy at each survey time point. For example, while persons younger than 55 years old accounted for 7% of total US deaths at the time of the July and August surveys, respondents estimated that they accounted for 43% of total deaths. In addition, while the proportion of COVID-19 hospitalizations that occurred in persons younger than 55 years old was 38%, respondents in the September and October surveys reported that this population accounted for 46%. The mortality rate of patients hospitalized with COVID-19 was estimated by respondents in the November survey to be 25% compared to an actual rate of 12%. The proportion of patients hospitalized after being infected with COVID-19 was estimated by respondents in the December survey to be 34% compared to an actual proportion of 12%.

### Association Between COVID-19 Health Risk Perceptions and Household Isolation

**Figure 2:**
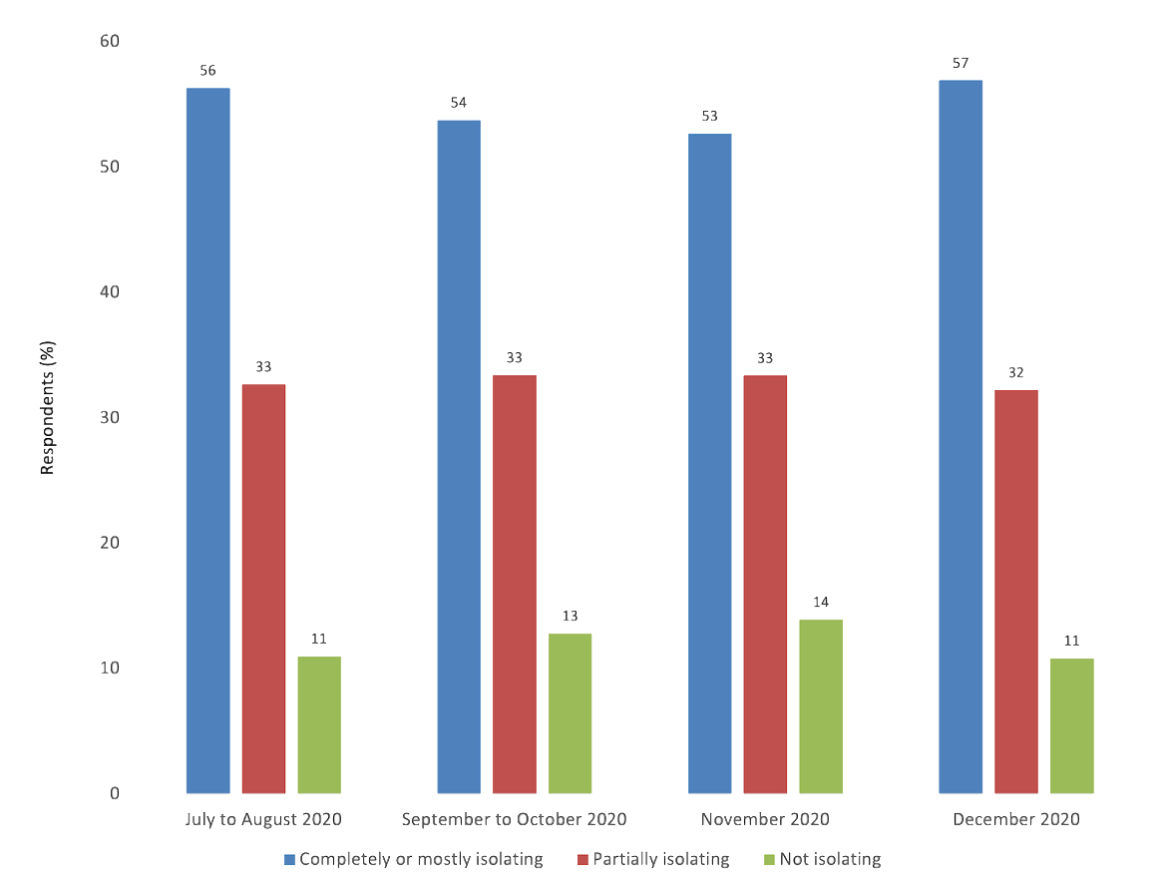
Rates of household isolation during the COVID-19 pandemic.

The proportion of respondents living in self-reported household isolation did not vary substantially over time, ranging from 53% to 57% (Figure 2). Overestimating the proportion of death or hospitalizations from COVID-19 occurring in people under 55 years old was associated with a significantly increased likelihood of self-reported household isolation (Table 2). Overestimating the likelihood of death or hospitalization was also associated with a significantly increased likelihood of self-reported household isolation. Excluding respondents who underestimated risk modestly attenuated the results, but all associations between misperceptions and social isolation remained significant (Table S1 in Multimedia Appendix 2). In the Gallup Panel, adults living in self-reported household isolation reported higher rates of loneliness (Figure S1 in Multimedia Appendix 2).

**Table 2:** Effect of misperceptions (ie, overestimation) of risk^a^ on the likelihood of living in self-reported household isolation for each time period.

Misperceptions	Likelihood of living in social isolation										
	July to August 2020			September to October 2020			November 2020			December 2020	
	Difference in % (95% CI)	*P* value	Difference in % (95% CI)		*P* value	Difference in % (95% CI)		*P* value	Difference in % (95% CI)		*P* value
Misperception about deaths^b^	7.7 (5.3-10.1)	<.001	N/A^c^		N/A	N/A		N/A	N/A		N/A
Misperception about hospitalizations^d^	N/A	N/A	5.6 (3.6-7.6)		<.001	N/A		N/A	N/A		N/A
Misperception about hospital mortality^e^	N/A	N/A	N/A		N/A	9.9 (6.9-12.8)		<.001	N/A		N/A
Misperception about hospitalization risk^f^	N/A	N/A	N/A		N/A	N/A		N/A	11.8 (8.7-14.9)		<.001

^a^Adjusted marginal effect of misperception (ie, overestimation) of risk.

^b^Misperception about proportion of COVID-19 deaths attributable to persons younger than 55 years old.

^c^N/A: not applicable; questions about misperception were only asked at specific time points.

^d^Misperception about proportion of COVID-19 hospitalizations attributable to persons younger than 55 years old.

^e^Misperception about proportion of patients hospitalized with COVID-19 who die.

^f^Misperception about hospitalization risk if infected with COVID-19.

### Other Characteristics Associated With Household Isolation

Characteristics associated with self-reported household isolation from the July and August 2020 surveys and persisting in the December 2020 survey included younger age (18 to 39 years), having a serious medical condition, having a household member with a serious medical condition, and identifying as a Democrat (Table 3). Being Black or Hispanic was associated with a higher likelihood of social isolation in July and August, but this relationship was not present in December. Reporting a higher income (>US $48,000) was associated with a lower likelihood of social isolation in July and August, but this relationship had largely waned by December.

**Table 3:** Association of misperceptions (ie, overestimation) about COVID-19 health risks with preferences for living in self-reported household isolation.

Characteristics		July to August 2020		September to October 2020			November 2020			December 2020	
		aOR^a^ (95% CI)	*P* value	aOR (95% CI)	*P* value	aOR (95% CI)		*P* value	aOR (95% CI)		*P* value
**Misperceptions about COVID-19 health risks**											
	Misperception about deaths^b^	1.38 (1.25-1.53)	<.001	N/A^c^	N/A	N/A		N/A	N/A		N/A
	Misperception about hospitalizations^d^	N/A	N/A	1.27 (1.17-1.38)	.001	N/A		N/A	N/A		N/A
	Misperception about hospital mortality^e^	N/A	N/A	N/A	N/A	1.52 (1.34-1.72)		.001	N/A		N/A
	Misperception about hospitalization risk^f^	N/A	N/A	N/A	N/A	N/A		N/A	1.65 (1.45-1.88)		<.001
**Age group (years)**											
	18-39 (reference)	1.00	—^g^	1.00	—	1.00		—	1.00		—
	40-64	0.73 (0.67-0.79)	.001	0.89 (0.80-0.98)	.01	0.76 (0.66-0.88)		<.001	0.84 (0.73-0.96)		.01
	≥65	0.88 (0.79-0.97)	.01	0.93 (0.83-1.05)	.25	0.76 (0.64-0.90)		.002	0.98 (0.82-1.17)		.83
**Female**											
	No (reference)	1.00	—	1.00	—	1.00		—	1.00		—
	Yes	0.96 (0.90-1.04)	.32	0.96 (0.88-1.04)	.33	0.86 (0.76-0.98)		.02	1.02 (0.90-1.15)		.78
**Race or ethnic group**											
	White (reference)	1.00	—	1.00	—	1.00		—	1.00		—
	Black	1.13 (1.00-1.26)	.04	1.44 (1.25-1.66)	<.001	1.33 (1.09-1.62)		.006	0.97 (0.79-1.18)		.74
	Hispanic	1.35 (1.22-1.49)	<.001	1.27 (1.12-1.43)	<.001	1.29 (1.09-1.54)		.004	0.97 (0.82-1.16)		.76
	Other	1.34 (1.17-1.53)	<.001	1.50 (1.27-1.75)	<.001	1.67 (1.33-2.10)		<.001	1.24 (0.99-1.56)		.06
**Educational level**											
	8th grade or some high school (reference)	1.00	—	1.00	—	1.00		—	1.00		—
	High school graduate	0.95 (0.79-1.15)	.59	1.00 (0.81-1.25)	.97	0.55 (0.41-0.75)		<.001	0.70 (0.50-0.99)		.04
	Some college or college graduate	1.00 (0.83-1.21)	.99	1.01 (0.81-1.26)	.95	0.64 (0.47-0.87)		.004	0.84 (0.60-1.19)		.32
**Serious medical condition**											
	No (reference)	1.00	—	1.00	—	1.00		—	1.00		—
	Yes, in respondent	2.15 (1.98-2.35)	<.001	2.26 (2.04-2.50)	<.001	1.78 (1.53-2.06)		<.001	1.85 (1.60-2.15)		<.001
	Yes, in household member	1.49 (1.35-1.64)	<.001	1.70 (1.51-1.91)	<.001	1.26 (1.07-1.50)		.007	1.34 (1.14-1.58)		<.001
	Yes, in respondent and household member	1.66 (1.45-1.91)	<.001	1.89 (1.61-2.23)	<.001	1.43 (1.14-1.79)		.002	1.73 (1.39-2.15)		<.001
**Political preference**											
	Democrat (reference)	1.00	—	1.00	—	1.00		—	1.00		—
	Republican	0.64 (0.59-0.70)	<.001	0.68 (0.62-0.76)	<.001	0.57 (0.50-0.67)		<.001	0.54 (0.47-0.63)		<.001
	Independent	0.72 (0.66-0.78)	<.001	0.79 (0.71-0.88)	<.001	0.75 (0.64-0.88)		<.001	0.76 (0.65-0.89)		<.001
	Other party	0.72 (0.61-0.84)	<.001	0.68 (0.56-0.84)	<.001	0.70 (0.52-0.95)		.02	0.67 (0.51-0.87)		.004
**Household income (US $)**											
	24,000 (reference)	1.00	—	1.00	—	1.00		—	1.00		—
	24,000-47,999	0.90 (0.81-1.01)	.07	1.02 (0.90-1.16)	.72	0.85 (0.71-1.01)		.07	0.96 (0.80-1.15)		.65
	48,000-89,999	0.80 (0.72-0.89)	<.001	0.92 (0.81-1.05)	.21	0.90 (0.75-1.08)		.24	0.98 (0.82-1.18)		.85
	≥90,000	0.89 (0.79-1.00)	.04	1.09 (0.95-1.26)	.23	1.11 (0.90-1.37)		.31	1.24 (1.01-1.53)		.04
**Married**											
	No (reference)	1.00	—	1.00	—	1.00		—	1.00		—
	Yes	0.99 (0.92-1.07)	.80	0.97 (0.89-1.06)	.54	1.04 (0.91-1.18)		.61	1.01 (0.88-1.15)		.92
**Live in rural area**											
	No (reference)	1.00	—	1.00	—	1.00		—	1.00		—
	Yes	0.93 (0.82-1.04)	.20	0.86 (0.75-0.99)	.04	0.95 (0.78-1.15)		.61	0.93 (0.76-1.13)		.45
Deaths per capita due to COVID-19		1.00 (0.99-1.00)	.79	1.01 (1.00-1.02)	.18	1.00 (0.99-1.02)		.54	1.00 (0.99-1.01)		.92

^a^aOR: adjusted odds ratio.

^b^Misperception about proportion of COVID-19 deaths attributable to persons younger than 55 years old.

^c^N/A: not applicable; questions about misperception were only asked at specific time points.

^d^Misperception about proportion of COVID-19 hospitalizations attributable to persons younger than 55 years old.

^e^Misperception about proportion of patients hospitalized with COVID-19 who die.

^f^Misperception about hospitalization risk if infected with COVID-19.

^g^*P* values cannot be calculated for reference values.

## Discussion

Adverse mental and emotional health effects of the COVID-19 pandemic are an increasingly recognized public health challenge. Risk misperceptions about COVID-19 may be exacerbating this challenge. Using Franklin Templeton-Gallup Economics of Recovery Study surveys from July to December 2020, we found that respondents consistently overestimated health risks associated with COVID-19, as measured by four different questions assessing COVID-19 morbidity and mortality. Overestimation of risk was consistently associated with greater self-reported household isolation, which may have adverse emotional and mental effects similar to quarantining [5,7]. These findings are relevant to policy interventions for social isolation and loneliness because they suggest that more accurate public understanding of risk would yield an optimal balance between health precautions and healthy social interactions.

While social isolation and loneliness have often been considered health risks for older adults, prior research has shown that COVID-19–related emotional and mental health harms are disproportionately borne by younger adults [3,17]. A June 2020 CDC survey reported that approximately twice as many respondents seriously considered suicide in the previous 30 days compared to US adults in 2018 when asked about the previous 12 months (10.7% versus 4.3%) [8]. The highest rates of suicidal ideation were reported by persons aged 18 to 24 years. We found that self-reported household isolation was most common among persons aged 18 to 39 years, a finding that likely contributes to the high rates of emotional distress reported in this population during the pandemic. The analysis of the Gallup Panel, which included detailed questions about emotional health, demonstrated that US adults reporting household isolation also reported higher rates of loneliness. The disproportionate burden on young people also raises concerns about long-term health and economic consequences.

Suicidal ideation among young adults is a concern because it is associated with a markedly increased risk of suicide plan and attempt, particularly during the first year after onset of ideation [18]. Among persons aged 18 to 25 years who participated in the 2009 to 2015 National Surveys on Drug Use and Health, the 12-month prevalence of suicidal ideation increased from 6.1% to 8.3% [19]. However, over this time period, receipt of mental health care was unchanged for most suicidal young adults and declined slightly among young adults without health insurance. This combination of trends may exacerbate the effects of worsening mental and emotional health during the COVID-19 pandemic.

Respondents who had a serious medical condition or lived with a household member with a serious medical condition were more likely to engage in self-reported household isolation. Based on public health messages about risk factors for an adverse COVID-19 outcome, this finding was anticipated. Our finding that political party was associated with differences in self-reported preventive health behavior for COVID-19 has been confirmed in other work, including a survey study of 3000 American adults performed in March 2020 [20,21]. The partisan differences appear to also extend to policy preferences in response to COVID-19 [20]. Younger adults also reported higher rates of household isolation compared to older adults. Because these characteristics were independently associated with self-reported household isolation, they identify groups that may benefit from targeted public health messaging, in instances when the anticipated benefits of stricter household isolation due to reduction in likelihood of transmission may be outweighed by the mental and emotional health costs. The corollary is that there may also be populations who would benefit from greater engagement in household isolation to reduce the risk of infection.

Our study has limitations. We asked respondents about household isolation over the previous 24 hours rather than over a longer period of time, which may have led to inaccuracies. However, the relatively stable distribution of self-reported household isolation from month to month suggests that a 24-hour recall period was informative. Although we measured self-reported household isolation, we could not quantify social isolation or loneliness for survey respondents in the Franklin Templeton-Gallup Economics of Recovery Study because we did not collect information about participation in group activities, social engagement with friends or relatives, or subjective experience of loneliness [22,23]. It is possible that some individuals who strictly avoided contact with people outside of their household experienced low levels of social isolation and loneliness, while others who did not isolate themselves experienced high levels of social isolation and loneliness. However, our analysis of Gallup Panel data showed that US adults who avoided contact with people outside their household also reported higher rates of loneliness. Furthermore, household isolation is analogous to quarantining, and research has shown that quarantining is associated with increased stress, anxiety, depression, fear, and detachment from other people [5,7].

Another limitation is that self-reporting bias may have affected the accuracy of our household isolation measure. However, the relatively stable distribution of self-reported household isolation across study periods suggests that this bias was minimal. In addition, the questions we used to assess risk perceptions changed over time, which precluded direct comparisons of risk perception between time periods. Another factor that further complicated measurement of COVID-19 health risks and the likelihood of reporting household isolation is that COVID-19 case levels varied during our study period. Our regression models adjusted for per capita COVID-19 cases at the county level, but there could be confounding effects from other pandemic factors that varied over time. Furthermore, despite differences in our survey questions related to COVID-19 risk perception, these questions consistently probed beliefs about hospitalization and mortality risk, and our finding of an association between risk overestimation and self-reported household isolation was consistent, despite the changing questions.

In conclusion, survey respondents overestimated several health risks associated with COVID-19, and this overestimation was associated with a respondent’s decision to avoid contact with people outside the household. This relationship was consistent from July to December. Harms to emotional and mental well-being experienced by US adults during the COVID-19 pandemic may be mitigated by addressing risk misconceptions and attenuating household isolation preferences that exceed public health guidelines.

## Appendix

Multimedia Appendix 1 Study survey.

Multimedia Appendix 2 Effect of misperceptions on the likelihood of living in household isolation and emotional distress by degree of household isolation and age.

## Abbreviations

CDC: Centers for Disease Control and Prevention
COVID-NET: COVID-19–Associated Hospitalization Surveillance Network
UCLA: University of California, Los Angeles
