# Supplementary material for: Association of COVID-19 Risk Misperceptions With Household Isolation in the United States: Survey Study
Source: JMIR Form Res. 2021 Aug 30;5(8):e30164. doi: 10.2196/30164 (PMC8407438; doi:10.2196/30164)
Supplement: Multimedia Appendix 1 [file formative_v5i8e30164_app1.docx]

**Multimedia Appendix 1**

**Survey Questions**

All Survey Time Points: Household Isolation

- Next, thinking about everything you have done in the past 24 hours, which of the following comes closest to describing your in-person contact with people outside your household?

1. Completely isolated yourself, having no contact with people outside your household
2. Mostly isolated yourself, having very little contact with people outside your household
3. Partially isolated yourself, having some contact with people outside your household
4. Isolated yourself a little, still having a fair amount of contact with people outside your household
5. Did not make any attempt to isolate yourself from people outside your household

All Survey Time Points in Gallup Panel: Loneliness

- Did you experience the following feelings during A LOT OF THE DAY yesterday?

1 Yes

2 No

- 1. Enjoyment
  2. Worry
  3. Sadness
  4. Stress
  5. Anger
  6. Happiness
  7. Boredom
  8. Loneliness
  9. Depression (added 5.11.2020)
  10. Anxiety (added 5.11.2020)
  11. Isolation (added 12.14.2020)

All Survey Time Points: Measures Clinical Risk Factors

- Do you or does anyone else in your household have a medical condition such as diabetes, heart disease, lung disease or something else that is considered high risk for serious complications from the coronavirus? (Responses: Yes, I do; Yes, another household member; Yes, both me and another household member; No)

July and August Surveys: Measures Misperceptions

- There have been 114,699 (140,906 ) deaths from COVID-19 as of June 13, 2020 (July 21, 2020). What percentage of deaths come from people in each of the following age groups, according to data from the Centers for Disease Control? Please provide your best estimate from 0% to 100% for each group. Your responses should add up to 100%. (Responses: Age 24 and below; Age 25-34**;** Age 35-44; Age 45-54; Age 55-64**;** Age 65 and older**)**

October Survey: Measures Misperceptions

- There have been 396,355 hospitalizations due to COVID-19 as of Sept. 21, 2020. What percentage of hospitalizations come from people in each of the following age groups, according to data from the COVID Tracking Project? Please provide your best estimate from 0% to 100% for each group. Your responses should add up to 100%.

November Survey: Measures Misperceptions

- There have been 443,777 hospitalizations due to COVID-19 as of October 22, 2020. Out of those hospitalized by COVID-19, what percentage do you think have ultimately died?

December Survey: Measures Misperceptions

- As far as you know, what percentage of people who have been infected by the coronavirus needed to be hospitalized?
