# Supplementary material for: Association of COVID-19 Risk Misperceptions With Household Isolation in the United States: Survey Study
Source: JMIR Form Res. 2021 Aug 30;5(8):e30164. doi: 10.2196/30164 (PMC8407438; doi:10.2196/30164)

**Multimedia Appendix 2**

Table S1. Effect of misperceptions (overestimating risk) on the likelihood of living in household isolation

|  |  |  |  |  |  |  |  |  |  |  |  |  |  |  |  |  |
| --- | --- | --- | --- | --- | --- | --- | --- | --- | --- | --- | --- | --- | --- | --- | --- | --- |
|  | July to August 2020 | | | | September to October 2020 | | | | November 2020 | | | | December 2020 | | | |
|  | Difference in Percent (95% CI) | | | P value | Difference in Percent (95% CI) | | | P value | Difference in Percent (95% CI) | | | P value | Difference in Percent (95% CI) | | | P value |
| Living in household isolation |  |  |  |  |  |  |  |  |  |  |  |  |  |  |  |  |
| Misperception about deaths* | 7.7 | (5.3- | 10.1) | <0.001 |  |  |  |  |  |  |  |  |  |  |  |  |
| Misperception about hospitalizations† |  |  |  |  | 5.6 | (3.6- | 7.6) | <0.001 |  |  |  |  |  |  |  |  |
| Misperception about hospital mortality∆ |  |  |  |  |  |  |  |  | 9.9 | (6.9- | 12.8) | <0.001 |  |  |  |  |
| Misperception about hospitalization risk‡ |  |  |  |  |  |  |  |  |  |  |  |  | 11.8 | (8.7- | 14.9) | <0.001 |
|  |  |  |  |  |  |  |  |  |  |  |  |  |  |  |  |  |

Note: Adjusted marginal effect of misperception (overestimation) of risk. Regression model excludes patients who underestimated risk

Abbreviations: CI, confidence interval; OR, odds ratio

* Misperception about proportion of COVID-19 deaths attributable to persons younger than 55 years old

† Misperception about proportion of COVID-19 hospitalizations attributable to persons younger than 55 years old

∆ Misperception about proportion of patients hospitalized with COVID-19 who die

‡ Misperception about hospitalization risk if infected with COVID-19

Figure S1. Emotional distress by degree of household isolation (completely, mostly, partially, little, and none) and age


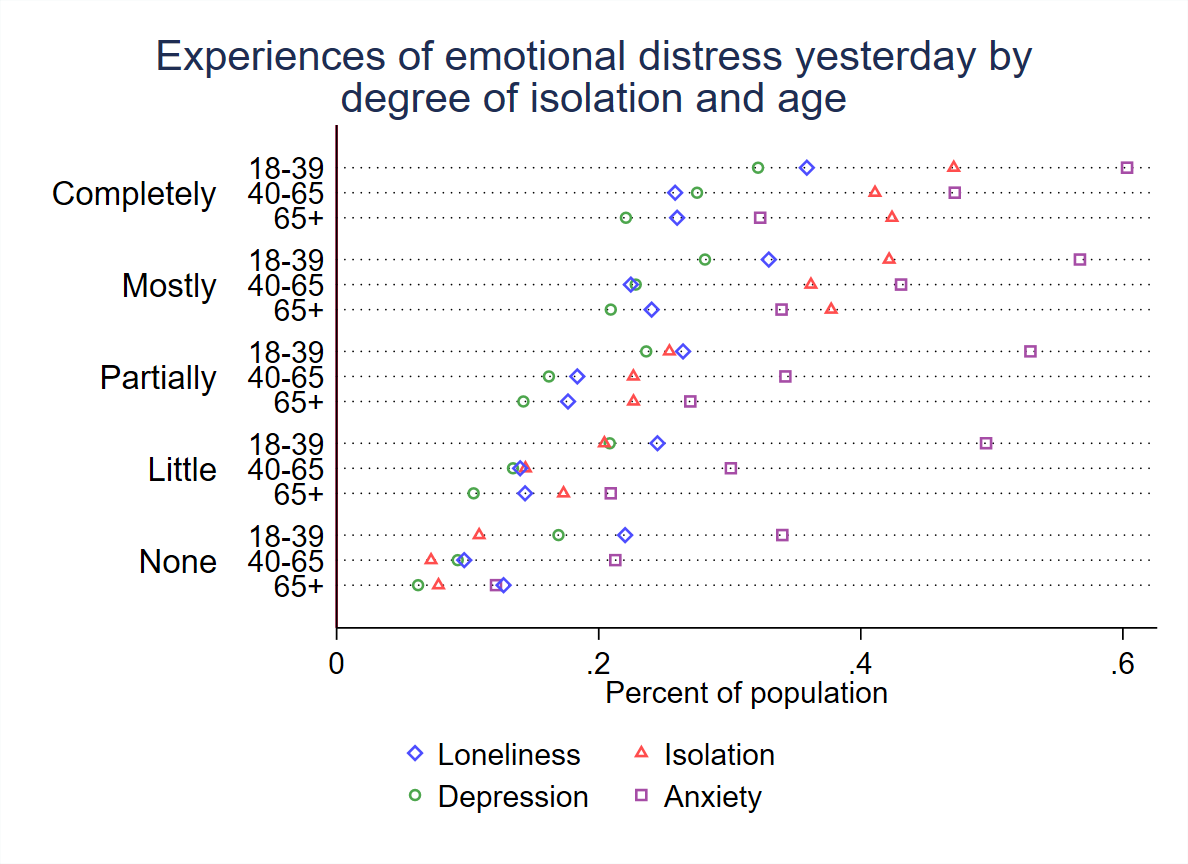

Supplement: Multimedia Appendix 2 [file formative_v5i8e30164_app2.docx]
